# Predictors of Clinically Significant Prostate Cancer in Patients with PIRADS Categories 3–5 Undergoing Magnetic Resonance Imaging-Ultrasound Fusion Biopsy of the Prostate

**DOI:** 10.3390/jcm12010156

**Published:** 2022-12-25

**Authors:** Stanisław Szempliński, Hubert Kamecki, Małgorzata Dębowska, Bartłomiej Zagożdżon, Mateusz Mokrzyś, Marek Zawadzki, Roman Sosnowski, Andrzej Tokarczyk, Sławomir Poletajew, Piotr Kryst, Łukasz Nyk

**Affiliations:** 1 Second Department of Urology, Centre of Postgraduate Medical Education, 01-809 Warsaw, Poland; 2 Medyczne Centrum Diagnostyczne Voxel, 00-671 Warsaw, Poland; 3 Nałęcz Institute of Biocybernetics and Biomedical Engineering, Polish Academy of Sciences, 02-109 Warsaw, Poland; 4 Department of Computational Oncology, Maria Skłodowska-Curie National Research Institute of Oncology, 02-781 Warsaw, Poland; 5 Department of Urology, St. Anna Hospital, 05-500 Piaseczno, Poland; 6 Department of Urogenital Cancer, Maria Skłodowska-Curie National Research Institute of Oncology, 02-781 Warsaw, Poland

**Keywords:** detection rate, multiparametric magnetic resonance imaging, positive predictive value, prostate imaging reporting and data system version 2, prostate cancer, targeted biopsy

## Abstract

Prostate biopsy is recommended in cases of positive magnetic resonance imaging (MRI), defined as Prostate Imaging Reporting and Data System (PIRADS) category ≥ 3. However, most men with positive MRIs will not be diagnosed with clinically significant prostate cancer (csPC). Our goal was to evaluate pre-biopsy characteristics that influence the probability of a csPC diagnosis in these patients. We retrospectively analyzed 740 consecutive men with a positive MRI and no prior PC diagnosis who underwent MRI-ultrasound fusion biopsies of the prostate in three centers. csPC detection rates (CDRs) for each PIRADS category were calculated. Patient, disease, and lesion characteristics were studied for interdependencies with the csPC diagnosis. The CDR in patients with PIRADS categories 3, 4, and 5 was 10.5%, 30.7%, and 54.6%, respectively. On both uni- and multivariable regression models, older age, being biopsy-naïve, prostate specific antigen ≥ 10 ng/mL, smaller prostate volume, PIRADS > 3, a larger maximum lesion size, a lesion in the peripheral zone, and a positive digital rectal examination were associated with csPC. In this large, multicenter study, we provide new data regarding CDRs in particular PIRADS categories. In addition, we present several strong predictors that further alter the risk of csPC in MRI-positive patients. Our results could help in refining individual risk assessment, especially in PIRADS 3 patients, in whom the risk of csPC is substantially low.

## 1. Introduction

Prostate cancer (PC) is the second most commonly diagnosed male malignancy [1], with mortality reaching approximately 375,000 annual deaths worldwide [2]. The likelihood of unfavorable outcomes strongly depends on individual cancer pathology, which has led to the development of the concept of clinically significant PC (csPC), which, contrary to low-risk PC, which is eligible for active surveillance, should be managed with active treatment. Many strategies have been aimed at tailoring the overall PC diagnosis yield to csPC cases only, with current data showing that the rates of men diagnosed with low-risk PC are on a downtrend [3]. The core initial risk stratification tool is magnetic resonance imaging (MRI) of the prostate and assessment with the Prostate Imaging Reporting and Data System (PIRADS) [4], with MRI-guided needle biopsies of the prostate allowing for the most accurate assessment of tumor pathology [5]. As recommended by the European Association of Urology, a prostate biopsy should be performed in cases of positive MRI, defined as PIRADS category 3 or higher [6].

However, contemporary data shows that most patients with a positive MRI who undergo prostate biopsy will not be diagnosed with csPC [7]. Thus, a positive MRI alone cannot be considered a strong predictor of a csPC diagnosis, and other risk factors should be pursued. In order to provide data that could help in refining individual risk assessment, the aim of this study was to retrospectively analyze a large cohort of MRI-positive patients who underwent MRI-ultrasound fusion biopsy of the prostate in order to provide csPC detection rates (cancer detection rates, CDR) for PIRADS categories 3, 4, and 5, as well as to study possible associations between specific patient or lesion characteristics and an increase in the risk of csPC.

## 2. Materials and Methods

We retrospectively analyzed consecutive patients without a prior history of PC who underwent MRI-ultrasound fusion biopsies of the prostate at three centers, including one university hospital, between March 2018 and October 2021. Data were collected from medical patient records and included: age, previous medical history, pre-biopsy prostate-specific antigen (PSA) level, MRI report, biopsy procedure report, and pathology report. The study included patients with PIRADS category 3 or higher. Patients with incomplete data were excluded from the study.

### 2.1. MRI-Ultrasound Fusion Biopsy

An MRI was performed either at our institutions or externally, with external studies having been reviewed by an institutional radiologist in case of ambiguities. PIRADS version 2.0 or 2.1 was used in all cases. All biopsies were performed with the KOELIS Trinity MRI/US OBT Fusion^®^ system, using either a transperineal or transrectal approach. Two experienced urologists, without the assistance of another physician, performed all the procedures at the three participating centers. A digital rectal examination (DRE) was carried out and recorded just before the procedure. The selection of the biopsy approach (transperineal or transrectal) was primarily indicated by individual urologist expertise, with the transperineal approach being preferred in order to reduce the risk of infectious complications. Transperineal biopsies were performed with the aid of the KOELIS Full Grid^TM^ device. Every biopsy included cores targeted at all the PIRADS ≥ 3 lesions identified in the MRI report. The number of targeted cores was never less than 3 per lesion, as defined by the policy the urologists adhered to. A greater number of targeted cores might have been taken if deemed necessary by the urologist. Occasionally, the biopsy may have also included additional cores targeted at lesions not identified in the report but considered suspicious by the performing urologist or the reviewing radiologist. In all biopsy-naïve patients, systematic cores were included. In patients with a previous negative biopsy, the addition of systematic cores was at the discretion of the urologist. Systematic cores, if included, did not cover the regions subject to targeted biopsy. The number of systematic cores was at the discretion of the urologist, being dependent predominantly on the lesion location and prostate volume. All the specimens were assessed by institutional pathologists. All the pathologists were specialists in urogenital cancer and adhered to the International Society of Urological Pathology (ISUP) guidelines.

### 2.2. Definitions

We defined a csPC diagnosis as the presence of grade group ≥ 2 cancer in either targeted or systematic cores. To represent a typical clinical scenario, we defined the highest PIRADS category as the highest category of a lesion as classified by the radiologist in the original MRI report, regardless of the performing urologist’s or reviewing institutional radiologist’s second opinion. The maximum lesion size was the size of the largest lesion in the highest PIRADS category in a patient. The number of cores was the total number of cores taken during the biopsy procedure.

### 2.3. Outcome Measurements and Statistical Analysis

Categorical and quantitative variables were calculated as numbers with percentages and medians (with interquartile ranges), respectively. The associations between categorical and continuous variables and a dependent variable were investigated using univariable and multivariable logistic regression models. The outcomes of logistic regression models were expressed as odds ratios (OR) with 95% confidence intervals (95% CIs). Results were considered statistically significant at a *p*-value < 0.05. Statistical analyses were performed using MATLAB R2021a (MathWorks, Natick, MA, USA) and R version 4.0.3 (R Foundation for Statistical Computing, Vienna, Austria).

## 3. Results

We identified 748 patients who met the inclusion criteria. Eight patients were excluded due to incomplete data. Eventually, 740 men were enrolled into the analyses. Data, including baseline patient and lesion characteristics, biopsy approach, median number of cores, and csPC diagnosis rate, are presented in Table 1.

**Figure 1:**
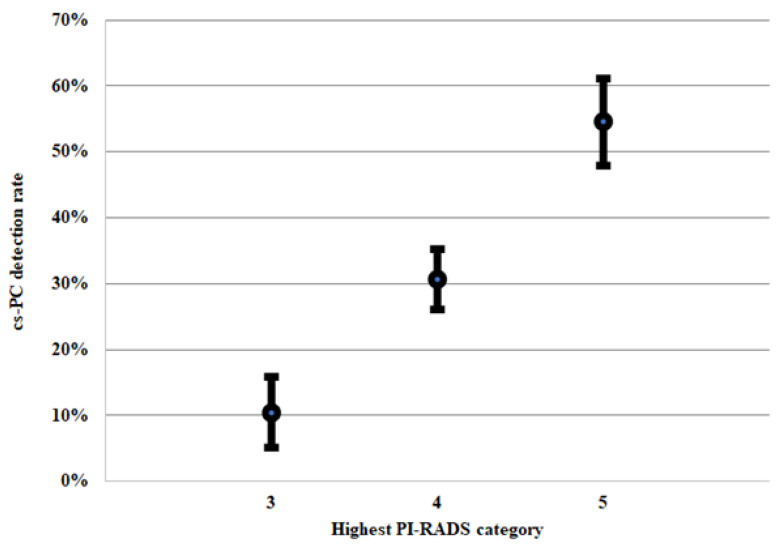
Detection rates of clinically significant prostate cancer (cs-PC) among patients with the highest Prostate Imaging Reporting and Data System (PIRADS) categories 3, 4, and 5. The whiskers represent 95% confidence intervals.

The CDR in patients with PIRADS categories 3, 4, and 5 was 10.5% (95% CI: 5.1–15.9%), 30.7% (95% CI: 26.1–35.2), and 54.6% (95% CI: 48.0–61.2%), respectively (Figure 1).

The type of biopsy approach (transrectal or transperineal) was not associated with the probability of a csPC diagnosis (CDRs: 37.6 vs. 33.7%, respectively, *p* = 0.75).

Concerning univariable analysis, older age, being biopsy-naïve, having a PSA level ≥ 10 ng/mL, a smaller prostate volume, a PIRADS category > 3, a larger maximum lesion size, a lesion located in the peripheral zone (PZ), and a positive digital rectal examination (DRE) were associated with an increased risk of csPC diagnosis (Table 2). All these variables were then included in a multivariable model, demonstrating a significant association with csPC (Table 2) as well.

Considering the possibility of observer bias when interpreting the DRE result, we developed another multivariable logistic regression model that did not include DRE; the statistical significance of the associations between the remaining variables and the dependent variable became even stronger (Table 3).

We performed a similar multivariable analysis limited to patients with the highest PIRADS category 3. In both models (DRE included and DRE excluded), only a smaller prostate volume demonstrated a statistically significant association with csPC (Table 4).

## 4. Discussion

We present one of the largest series of MRI-positive patients who underwent biopsies of the prostate. MRI-ultrasound fusion biopsy was performed in every case, and while any possible superiority of this technique over cognitive biopsy remains controversial, with trends toward improved CDRs remaining statistically insignificant in a meta-analysis [8], fusion biopsy may serve as an acceptable reference standard in terms of evaluating MRI diagnostic values. We consider this a strength of our study.

Interestingly, our cancer detection rates, especially for PIRADS categories 4 and 5, were significantly lower than the available data would suggest. In a meta-analysis by Mazzone et al., rates of csPC with PIRADS categories 4 and 5 were reported to be 40% (95% CI: 34–46%) and 69% (65–73%), respectively [7], which barely overlaps with our 95% CIs for these values. Oerther et al., in a meta-analysis limited to studies in which PIRADS v. 2.1 was adopted, demonstrated the CDRs to be even higher [9]. The large heterogeneity between studies reporting CDRs in MRI-positive patients undergoing prostate biopsy is a well-recognized issue [7]. We believe that the most probable explanation for the relatively low rate of csPC in our patients is the significant (44%) proportion of men with a prior biopsy history. As demonstrated in the results, these men were significantly less likely to be diagnosed with csPC than biopsy-naïve patients, which is in accordance with available evidence. A recent prospective study by Patel et al. [10] also demonstrated that being biopsy-naïve was a significant factor for csPC diagnosis in MRI-positive patients, which confirms the trends previously described in the literature [7].

Given the abovementioned discrepancies in reported CDRs between cohorts of MRI-positive patients, the role of factors other than the PIRADS category in altering the probability of a csPC diagnosis is unquestionable, and our aim was to provide evidence regarding these associations. Being biopsy-naïve has already been discussed above. Age was another predictor we evaluated. While Washino et al. demonstrated no significant association between age and csPC in their patients [11], most studies report older age to be a strong predictor of csPC diagnosis, independently of the PIRADS category [10,12]. Whether this finding represents the well-established association between older age and increased incidence of csPC [13] or age-related features possibly altering MRI interpretation [14], remains beyond the scope of these considerations.

Given that non-csPC may not lead to PSA level elevations independent of the contribution of benign prostate tissue [15], high PSA levels should serve as predictors of csPC. Several studies demonstrated that PSA density (PSAD) may increase the risk of csPC independently of PIRADS category [10,11,16]. We decided to analyze PSA and PV as separate predictors, considering the possible independent association between smaller PV and csPC [17,18]. On multivariable analyses, we demonstrated significant associations with csPC for both PSA > 10 ng/mL and smaller PV. Moreover, in PIRADS category 3 patients, only the smaller PV was associated with higher csPC rates. To our knowledge, this is the first study in which PV was considered an independent risk factor in a regression model. Our results suggest that the relationship between those parameters and prostate cancer biology may be much more complex than represented by a proportion (i.e., PSAD). Further studies are needed to provide deeper insight into the predictive values of PSA and PV in patients suspected of harboring csPC.

We demonstrated that a larger maximum lesion size was associated with a higher CDR. In analyses performed on the overall group, this larger maximum lesion size might have represented a higher PIRADS category, given that ≥15 mm in the maximal dimension of a lesion is a criterion for assigning PIRADS category 5 instead of 4. Furthermore, in the analysis limited to PIRADS category 3 patients, the association between maximum lesion size and csPC was non-significant. Given the low CDR in this subgroup of patients, a small sample size might have been a limitation. Nevertheless, Tan et al., in a study on men who underwent in-bore MRI-guided transrectal targeted prostate biopsy, demonstrated no significant difference in the median diameter of the lesion between patients with negative and positive biopsy findings [19]. The role of maximum lesion size, other than differentiating between PIRADS categories 4 and 5, in stratifying the risk of csPC in men with positive MRIs should be subject to further studies.

Available data suggests that the prostate zone may serve as an additional factor predictive of csPS in patients with a positive MRI [20]. In the overall group, we demonstrated that a lesion located in the PZ was strongly associated with a higher CDR. In the PIRADS category three subgroup, despite a high OR, the association was non-significant, possibly due to an insufficient sample size. However, Kim et al., in a study on PIRADS category three patients who underwent MRI-ultrasound fusion targeted biopsy, did demonstrate that PZ location was an independent predictor of csPC [21]. Felker et al. suggested that many men with PIRADS category 3 lesions in the transition zone (TZ) might not be considered candidates for biopsy due to low csPC probability [22]. Our results may serve as additional evidence helpful in the decision-making process for these patients.

Despite multiple limitations, DRE remains a simple and cost-effective tool in the initial assessment of patients suspected of PC. While offering a biopsy of the prostate based solely on a positive DRE may be considered controversial in many cases, the available evidence proves that a positive DRE is a very strong predictor of csPC in MRI-positive patients. Chang et al. demonstrated that positive DRE had 91% specificity for csPC in men with positive MRI and elevated PSA who underwent MRI-ultrasound fusion biopsy [23]. Omri et al. also reported higher CDR in men with positive DRE [24]. In our study group, based on the results of multivariable analysis, patients with a positive DRE had almost double the odds of being diagnosed with csPC. The association in the PIRADS category 3 subgroup was non-significant. Nevertheless, both the literature data and our results demonstrate that a biopsy of the prostate should be offered to every man with an MRI and DRE suggestive of a malignant tumor.

We decided to perform separate analyses in the subgroup of PIRADS category 3 patients, as the low CDR in these men encourages the identification of risk factors, allowing for the offering of a prostate biopsy only to patients with a significant probability of a csPC diagnosis. In the study by Kim et al., older age, PZ location, and higher PSAD were associated with csPC on multivariable analysis [21]. Sheridan et al. demonstrated older age, smaller PV, and positive DRE as risk factors for csPC in patients with PIRADS category 3 lesions [25]. Felker et al. suggested PSAD ≥ 0.15 ng/mL^2^ and an apparent diffusion coefficient (ADC) < 1000 mm^2^/s as criteria that would lead to a much higher yield for csPC in men with PIRADS category 3 TZ lesions [22]. Recently, Schoots et al., based on the results of a meta-analysis that included data from 3006 biopsy-naïve patients, suggested that a prostate biopsy should be performed in a patient with a PIRADS category 3 lesion in the case of a PSAD ≥ 0.1 [26]. In our study, we managed to demonstrate a significant association only for smaller PV. The non-significance of other factors may be explained both by a lack of association and by the small sample size discussed above. Further studies on large populations or meta-analyses of available data are paramount to establishing the best evidence-based strategies for men with PIRADS category 3 lesions.

While we are aware that the discussed risk factors for detection of csPC have already been evaluated in the literature, this is the first large-volume study using MRI-ultrasound fusion biopsy results as a reference in which this particular set of clinically relevant and easily assessed factors was incorporated into a regression model. Hence, our results may possibly serve as evidence useful for weighted clinical judgment in patients in whom the individual low probability of harboring csPC is considered against the risk of biopsy complications. In our study, we adopted a per-patient, not per-lesion strategy for data analysis. The meta-analysis by Mazzone et al. demonstrated that per-lesion-level analysis may lead to lower rates of csPC [7]. Even with the use of a reference MRI-ultrasound fusion technique, cores still may miss the malignant lesion due to technical targeting mistakes or MRI limitations in detecting multifocal disease, and the identification of men who would benefit from omitting systematic cores is currently infeasible [27]. The per-patient study design was aimed to represent a typical clinical scenario.

While the multicenter design of the study may be considered a strength, some possible limitations must be addressed. All the biopsies were performed using the same software and materials. Hence, we deemed the quality of the cores to be similar between the centers. While the significantly lower median number of cores taken at Center 3 (Table 1) may raise concerns, this did not translate into decreased CDR in this institution. Although all the specimens were assessed according to the ISUP guidelines, possible interobserver variability between institutional pathologists might have been a source of bias. No uniform review of specimens may be considered a limitation of the study. Additionally, the results of DRE might have varied largely between the clinicians performing a biopsy. However, in order to exclude a possible bias caused by heterogeneity with regard to DRE interpretation, we performed a sub-analysis of the data without including DRE results.

Combining patients who underwent transrectal and transperineal biopsy into one cohort may be considered a possible cause of bias, as the non-inferiority of the transrectal approach in terms of csPC detection is not well-established [28]. However, in our patients, the difference in CDRs between transrectal and transperineal cases was non-significant. The main limitation of our study is its retrospective design, which implies several drawbacks. The study is prone to selection bias, as we were unable to verify uniform criteria for patients being referred to biopsy, which might have been at the discretion of various external urologists, and depending on the individual clinical judgment of each external urologist, some patients at the same risk might have been offered different diagnostic strategies. There was no standardized biopsy protocol used for our patients, which could have significantly influenced the pathologic outcomes. We lack data in regard to the number of targeted versus systematic cores, which could be valuable in the analyses. Considering the possibly important role of a second opinion [29], no uniform review of MRI scans poses the study at risk of bias due to potential initial misinterpretations. Some data gaps might have been a source of bias. Nevertheless, patients without sufficient data were not included in the regression models. Including follow-up data in the study, especially in regard to possible re-biopsy or radical prostatectomy specimens, could also have influenced the diagnostic performance of the pre-biopsy MRI. In addition, analysis of several other factors, like family history, multifocality, or anatomic lesion location (base vs. mid-prostate vs. apex), could have provided deeper insight into the heterogeneity of CDR in MRI-positive patients.

## 5. Conclusions

Our large, multicenter, retrospective study exploring the csPC detection rates in MRI-positive patients undergoing MRI-ultrasound fusion biopsy of the prostate provides new data regarding the predictive values of particular PIRADS categories, with our values being slightly lower than the current literature would suggest. This study serves as another piece of evidence that the probability of a csPC diagnosis in patients with the PIRADS 3 category is substantially low, warranting further risk stratification prior to offering a biopsy.

We managed to investigate several factors that further increase the probability of a csPC diagnosis in patients with a positive MRI. Apart from a higher PIRADS category or lesion size, patients were more likely to be diagnosed with csPC in cases of older age, lower PV, positive DRE, a lesion located in the PZ, and being biopsy-naïve. The role of lower PV was especially significant in PIRADS category 3 patients. The results of this study may be helpful in the decision-making process for patients considered for prostate biopsy. Moreover, they point to important future research directions.

## Figures and Tables

**Table 1:** Characteristics of patients.

Characteristic	All Patients (n = 740)	Center 1 (n = 298)	Center 2 (n = 122)	Center 3 (n = 320)
Median age, years (IQR)	65 (60, 69)	64 (58, 69)	66 (62, 69)	65 (61, 69)
Biopsy-naïve (%)	416 (56.2)	165 (55.4)	56 (45.9)	195 (60.9)
Median PSA, ng/mL (IQR)	6.9 (4.9, 9.7)	7.0 (5.1, 10.0)	7.5 (5.4, 10.0)	6.2 (4.7–9.1)
Median PV, mL (IQR)	42.7 (33.1, 59.7)	41.0 (32.8, 55.9)	48.9 (37.3, 63.0)	42.4 (33.0, 60.0)
Median max. lesion size, mm (IQR)	13 (10, 17)	13 (10, 16)	14 (10, 18)	13 (9, 16)
PIRADS category (%)				
3	124 (16.8)	63 (21.1)	11 (9.0)	50 (15.6)
4	398 (53.8)	144 (48.3)	66 (54.1)	188 (58.8)
5	218 (29.5)	91 (30.5)	45 (36.9)	82 (25.6)
Lesion in the peripheral zone (%)	485 (69.6) ^a^	158 (62.0) ^a^	101 (82.8)	226 (70.1)
Positive DRE ^b^	131 (18.5) ^b^	56 (21.0) ^b^	13 (10.7)	62 (19.4)
Biopsy approach, n				
Transperineal	615 (83.1)	182 (61.1)	113 (92.4)	320 (100.0)
Transrectal	125 (16.9)	116 (38.9)	9 (7.6)	0 (0.0)
Median number of cores (IQR)	11 (9, 15)	17 (16, 19)	19 (18, 23)	11 (9, 14)
Diagnosis of csPC (%)	254 (34.3)	114 (38.3)	35 (28.7)	105 (32.8)

IQR, interquartile range; PSA, prostate-specific antigen; PV—prostate volume; PIRADS—prostate imaging reporting and data system; DRE, digital rectal examination; csPC, or clinically significant prostate cancer. Center 1 was university-affiliated. ^a^ Data lacking for 43 patients, percentages calculated for known data. ^b^ Data lacking for 31 patients, percentages were calculated using known data.

**Table 2:** Association between clinically significant prostate cancer (csPC) diagnosis and other factors, DRE included.

	UVA vs. csPC OR (95% CI), *p*-Value	MVA vs. csPC OR (95% CI), *p*-Value
Age, years	1.05 (1.03–1.07), <0.001	1.05 (1.03–1.08), <0.001
Biopsy-naïve	1.42 (1.04–1.93), 0.027	1.57 (1.08–2.29), 0.017
PSA > 10 ng/mL	2.57 (1.81–3.65), <0.001	2.36 (1.53–3.64), <0.001
PV, mL	0.98 (0.98–0.99), <0.001	0.98 (0.97–0.98), <0.001
Max. lesion size, mm	1.07 (1.05–1.10), <0.001	1.05 (1.02–1.08), 0.001
PIRADS > 3	5.49 (3.02–9.10), <0.001	3.14 (1.63–6.05), 0.001
Lesion in PZ	2.05 (1.43–2.95), <0.001	1.86 (1.24–2.79), 0.003
Positive DRE	3.14 (2.12–4.63), <0.001	1.74 (1.12–2.70), 0.014

UVA, univariable analysis; MVA, multivariable analysis (logistic regression model, n = 697); OR, odds ratio; CI, confidence interval; PSA, prostate-specific antigen; PIRADS, prostate imaging reporting, and data system; PZ, peripheral zone; DRE, digital rectal examination.

**Table 3:** Association between clinically significant prostate cancer (csPC) diagnosis and other factors, digital rectal examination (DRE) excluded.

	MVA vs. csPC OR (95% CI), *p*-Value
Age, years	1.05 (1.03–1.08), <0.001
Biopsy-naïve	1.69 (1.17–2.44), 0.005
PSA > 10 ng/mL	2.43 (1.58–3.75), <0.001
PV, mL	0.98 (0.97–0.98), <0.001
Max. lesion size, mm	1.06 (1.03–1.09), <0.001
PIRADS > 3	3.27 (1.69–6.31), <0.001
Lesion in PZ	1.95 (1.30–2.92), 0.001

MVA, multivariable analysis (logistic regression model, n = 697); OR, odds ratio; CI, confidence interval; PSA, prostate-specific antigen; PIRADS, prostate imaging reporting, and data system; PZ, peripheral zone; DRE, digital rectal examination.

**Table 4:** Association between clinically significant prostate cancer (csPC) diagnosis and other factors in patients with the highest PIRADS category 3.

	MVA vs. csPC, DRE Included OR (95% CI), *p*-Value	MVA vs. csPC, DRE Excluded OR (95% CI), *p*-Value
Age, years	1.08 (0.98–1.19), NS	1.08 (0.98–1.19), NS
Biopsy-naïve	0.81 (0.20–3.39), NS	0.86 (0.21–3.43), NS
PSA > 10 ng/mL	1.69 (0.32–8.86), NS	1.46 (0.28–7.52), NS
PV, mL	0.94 (0.90–0.99), 0.019	0.94 (0.89–0.99), 0.017
Max. lesion size, mm	1.02 (0.88–1.19), NS	1.03 (0.88–1.19), NS
Lesion in PZ	3.17 (0.58–17.37), NS	3.09 (0.58–16.34), NS
Positive DRE	2.93 (0.42–20.31), NS	N.A.

MVA, multivariable analysis (logistic regression model, n = 114); OR, odds ratio; CI, confidence interval; PSA, prostate specific antigen; PIRADS, prostate imaging reporting and data system; PZ, peripheral zone; DRE, digital rectal examination; NS—nonsignificant; N.A.—not-applicable.

## Data Availability

The data analyzed in this study are available upon request from the corresponding author.
